# Effects of Melatonin on Anti-oxidative Systems and Photosystem II in Cold-Stressed Rice Seedlings

**DOI:** 10.3389/fpls.2017.00785

**Published:** 2017-05-11

**Authors:** Qiao-Hong Han, Bo Huang, Chun-Bang Ding, Zhong-Wei Zhang, Yang-Er Chen, Chao Hu, Li-Jun Zhou, Yan Huang, Jin-Qiu Liao, Shu Yuan, Ming Yuan

**Affiliations:** ^1^College of Life Sciences, Sichuan Agricultural UniversityYa’an, China; ^2^College of Resources, Sichuan Agricultural UniversityChengdu, China

**Keywords:** melatonin, cold stress, reactive oxygen species, photosynthetic parameters, chlorophyll fluorescence

## Abstract

Melatonin (*N*-acetyl-5-methoxytryptamine) plays important role in multiple plant developmental processes and stress responses. We investigated the possible mediatory role of melatonin in growth, photosynthesis, and the response to cold stress in rice by using three different experiments: soaking seed; immersing roots, and spraying to leaves with 0, 20, or 100 μM melatonin. After 6 days of cold stress, the growth of rice seedlings was significantly inhibited, but this inhibition was alleviated by exogenous melatonin. Furthermore, exogenous melatonin pretreatment alleviated the accumulation of reactive oxygen species, malondialdehyde and cell death induced by cold stress. Melatonin pretreatment also relieved the stress-induced inhibitions to photosynthesis and photosystem II activities. Further investigations showed that, antioxidant enzyme activities and non-enzymatic antioxidant levels were increased by melatonin pretreatments. The treatment methods of seed soaking and root immersion were more effective in improving cold stress resistance than the spraying method. The results also indicated the dose-dependent response of melatonin on rice physiological, biochemical, and photosynthetic parameters.

## Introduction

Low temperature is one of the major abiotic stresses that limits crop growth, productivity, survival and geographical distribution of plants (Mishra et al., 2011; Hu et al., 2016), especially in temperate zones and high-elevation environments (Andaya and Mackill, 2003). Exposure of plants to cold tress results in changes in multiple physiological, biochemical, molecular and metabolic processes including alternations of membrane fluidity, enzyme activities and metabolism homeostasis (Bajwa et al., 2014; Hu et al., 2016). Plants growth at low temperature also lead to oxidative stress through increasing reactive oxygen species (ROS), such as hydrogen peroxide, superoxide anion, and hydroxyl radicals (Taşgın et al., 2006; Hu et al., 2016). The accumulation of ROS causes peroxidation of lipids and oxidation of proteins within cells, resulting in inhibition to plant growth (Cao et al., 2015; Nahar et al., 2015). Therefore, to prevent the oxidative injury induced by ROS, plants have evolved an complex antioxidant system including enzymatic antioxidants such as superoxide dismutase (SOD), peroxidase (POD), catalase (CAT) and non-enzymatic antioxidants such as glutathione and proline (Erdal, 2012; Erdal et al., 2015; Ghaderian et al., 2015; Chen et al., 2016).

Melatonin (*N*-acetyl-5-methoxytryptamine), a low molecular weight organic compound, is produced by the vertebrate pineal secretory (Pape and Lüning, 2006; Tan et al., 2012). It was first isolated from bovine pineal glands (Lerner et al., 1958), act as a animals hormone, which is involved in many physiological processes including circadian rhythm (Hardeland et al., 2012), immunological enhancement (Calvo et al., 2013), antioxidative processes (Galano et al., 2011; Reiter et al., 2013), sleep physiology (Jan et al., 2009), seasonal reproductive physiology (Barrett and Bolborea, 2012), sexual behavior and temperature homeostasis (Arnao and Hernández-Ruiz, 2015). Melatonin has also been found in almost all forms of organisms, like protists, invertebrates, algae, fungi, and bacteria (Pöggeler et al., 1991; Balzer and Hardeland, 1992; Tilden et al., 1997; Sprenger et al., 1999; Hardeland and Pöggeler, 2003). Dubbels et al. (1995) and Hattori et al. (1995) first identified melatonin in plants. Thereafter, further studies showed that melatonin was exist in various plant species (Manchester et al., 2000; Ramakrishna et al., 2012; Arnao and Hernández-Ruiz, 2013; Vitalini et al., 2013). Melatonin in plants may be involved in multiple developmental processes, including root architecture (Zhang et al., 2014), circadian rhythms regulation, regulation of flower development, photosynthesis promotion, regulation of fruit ripening, chlorophyll preservation (Arnao and Hernández-Ruiz, 2009; Tan et al., 2012), leaf senescence (Byeon et al., 2012; Wang et al., 2013a) and alleviation of oxidative damages through effectively scavenging ROS and reactive nitrogen species (RNS) (Arnao and Hernández-Ruiz, 2015). In addition, melatonin has been reported to protect plants against multiple abiotic stresses, such as salt, cold, drought and pathogen infections (Yin et al., 2013; Bajwa et al., 2014; Wang et al., 2014; Shi et al., 2015a,b,c). Recently, Shi et al. (2015a) found that cold stress activated the synthesis of melatonin in bermudagrass, and exogenous melatonin improved its cold stress tolerance by scavenging ROS directly and improving the antioxidative enzymes activities. However, the underlying physiological and molecular mechanism of melatonin in the induction of tolerance to cold stresses remains unclear in plants, and the effects of different melatonin-application method have not been compared. In this study, exogenous melatonin treatment was applied to investigate the potential roles of melatonin in rice response to cold stress. In addition, the effects of three different modes of melatonin application on physiological processes have been investigated. The first method was soaking seed with different concentrations of melatonin (Liu et al., 2015). The second method was immersing roots in Hoagland’s nutrient solution containing different concentrations of melatonin (Shi et al., 2015a). The third method was spraying leaves with different concentrations of melatonin solution (Hu et al., 2016). To our knowledge, these treatment methods have not been compared before. We investigated plant growth, ROS production, membrane lipid peroxide, chlorophyll fluorescence, photosynthetic parameters, antioxidant enzyme activities, and photosystem (II) PSII proteins after the melatonin pretreatment and the subsequent cold stress. The results could be helpful in understanding the physiological functions of melatonin in plants under low temperature stress.

## Materials and Methods

### Plant Materials and Growth Conditions

The seeds (*Oryza sativa* L. cv. DM You 6188) were sterilized with 3% (m/v) sodium hypochlorite for 10 min, washed with distilled water for five times and soaked in distilled water or melatonin (20 or 100 μM) for 36 h, then placed in Petri dishes with wetted filter paper, and germinated in the dark at 28°C in a growth chamber. After 4 days, the germinated seeds were transferred into soil with half strength Hoagland’s solution. The seedlings were maintained at a 14-h photoperiod, a photosynthetic photon flux density of 150 μmol m^-2^ s^-1^, day/night temperatures of 28/22°C and a relative humidity of 70%.

### Cold-Stress Treatment

Twelve-day-old seedlings were washed with tap water, and dried briefly with absorbent paper to remove surface water. There were three different ways for application of melatonin. The first method (I) was soaking seeds with 20 or 100 μM melatonin and watered with the Hoagland’s nutrient solution, and soaking seeds with water as a control. The second method (II) was that the rice seedlings were watered with the Hoagland’s solution containing 20 or 100 μM melatonin for 2 days. The seedlings were grown in the Hoagland’s solution as a control. The third method (III) was that the rice seedling leaves were sprayed every 4 h with 20 or 100 μM melatonin for 2 days. The control seedlings were sprayed with a similar solution without melatonin. After melatonin pretreatment, the 14-day-old seedlings were transferred to 12°C, and the control seedlings remain in day/night temperatures of 28/22°C. After 6-day treatment, the second leaves were used for the measurements, and all experiments were repeated at least three times.

### Extraction and Quantification of Melatonin by HPLC-MS/MS

Extraction of melatonin from rice was performed as described by Stürtz et al. (2011). The endogenous melatonin quantification was performed using liquid a chromatography (HPLC) system (1290 LC, Agilent, USA) couple to a mass spectrum (MS) system (6470 LC-MS/MS, Agilent, USA) according to Stürtz et al. (2011) with some modifications. MS–MS parameters were as follows: positive ion mode; turbo 1 speed, 100%; turbo 2 speed, 100%; sheath gas temperature, 300°C; sheath gas flow, 11.0 L/min; capillary current, 59 nA; capillary, 3368 V; MS 1 heater, 100°C; MS 2 heater, 100°C; rough vac, 9.91E-1 Torr; high vac, 3.60E-5 Torr; and m/z, 159.0. Separations were carried out on a 150 × 2.1 mm, 1.8 μm, Eldath RS-C18 column. Two different solvents were used in the mobile phase: solvent A (methanol) and solvent B (methanol with 0.1% formic acid, v/v), at a flow rate of 0.3 mL/min, injection volume of 1 μL and methanol was from 20 to 80%.

### Determination of Shoot and Root Dry Weight

After 6 days cold stress, shoots and roots were separated, then washed with tap water and rinsed twice with distilled water, gently wiped dry with a paper towel, and then were oven dried at 80°C to constant weight for DW determination, respectively.

### Determination of Leaf Relative Water Content (RWC) and Root Activity

The RWC was measured according to Cao et al. (2015). The second leaves were excised and their fresh mass (FM) was determined immediately, after floated on deionized water for 5 h, the saturated mass (SM) was recorded. Then the leaves were dried at 80°C to constant weight to measure their dry mass (DM). The RWC was calculated from the following equation:

RWC[%] =[(FM-DM)/(SM-DM)]×100.

Root activity was assessed by TTC (triphenyl tetrazolium chloride) method (Chen, 2003), and was expressed as the deoxidization ability (μg g^-1^h^-1^).

### Determination of Chlorophyll and Carotenoids Contents

Chlorophyll (Chl) content was measured as previously described Lichtenthaler and Wellburn (1983). Fresh leaves (0.1 g) were cut and homogenized with 20 mL of 80% (v/v) acetone in a pre-chilled mortar and pestle, then centrifuged at 8000 *g* for 10 min. The supernatant was separated and the absorbance was measured with a spectrophotometer (UV-1750, Shimadzu, Japan) at 663, 646 and 470 nm. The content of chlorophyll and carotenoids were calculated using the Lichtenthaler and Wellburn formula (Lichtenthaler and Wellburn, 1983) and expressed in mg g^-1^ FW.

### Determination of Electrolyte Leakage (EL) and Malondialdehyde (MDA)

Electrolyte leakage was determined using a electrical conductivity meter (DDS-309+, Chengdu, China) following Ning et al. (2015). The second leaves were taken and cut into 5 mm segments, weighted and placed in 50 mL capped polypropylene centrifuge tubes containing 25 mL distilled water. The initial electrical conductivity (EC1) of the bathing solutions was measured after the samples were incubated at 25°C for 24 h. Then, the samples and bathing solutions boiled in water for 30 min and measured electrical conductivity (EC2) again after cooling the solution to room temperature. EL was calculated using the formula: EL = EC1/ EC2 × 100.

The degree of membrane lipid peroxidation in leaves was estimated by MDA content. MDA was evaluated using thiobarbituric acid assay (Nahar et al., 2015). Fresh leaves (0.5 g) was homogenized in 5 mL of trichloroacetic acid (TCA) (5%, m/v) and centrifuged at 8000 *g* and 4°C for 10 min. 2 mL of the supernatant was mixed with the equal volume 0.5% thiobarbituric acid. The mixture was bathed at 95°C for 30 min and then instantly cooled on ice and centrifuged at 5000 *g* and 4°C for 10 min. The absorbance of the supernatant was recorded at 450, 532, and 600 nm, respectively. MDA content was calculated according to Nahar et al. (2015) and expressed as μmol g^-1^ FW.

### Determination of H_2_O_2_ and $O_{2}^{-}$

$O_{2}^{-}$ and H_2_O_2_ were visually detected in leaves with nitro blue tetrazolium (NBT) and 3,3-diaminobenzidine (DAB), respectively, as described previously method (Yang et al., 2004). The leaves were excised at the base and immersed into NBT (1 mg/mL) solution for 2 h or into DAB (0.5 mg/mL) solution for 12 h in the dark, then boiling in ethanol (90%, v/v) for 0.5–2 h to decolorize the leaves. At least five leaves were used for each staining.

The content of H_2_O_2_ was determined as described by Velikova et al. (2000). 0.5 g fresh leaves were homogenized with 5 mL 0.1% (m/v) TCA in an ice bath and centrifuged for 15 min at 12 000 × *g* and 4°C. 0.5 mL of the supernatant was added to 0.5 mL of 10 mM potassium phosphate buffer (pH 7.0) and 1 mL of 1 M KI. The absorbance of the mixture was recorded at 390 nm. Finally, a standard curve of H_2_O_2_ reagent was used to calculated the H_2_O_2_ content. The content of H_2_O_2_ was expressed as μmol g^-1^ FW.

Production of $O_{2}^{-}$ was estimated following Nahar et al. (2015) with some modification. 0.2 g fresh leaves was homogenized with 2 mL 65 mM phosphate buffer (pH 7.8), and centrifuged at 10000 *g* and 4°C for 10 min. Then, 0.5 mL supernatant was mixed with 0.5 mL 65 mM phosphate buffer (pH 7.8) and 0.1 mL 10 mM hydroxylamine hydrochloride. After 20 min incubated at 25°C, the solution was added to 1 mL 17 mM sulfanilamide and 1 mL 7 mM naphthylamine and incubated at 25°C for 20 min. Absorbance was recorded at 530 nm. The formation $O_{2}^{-}$ was calculated using a standard curve of a NaNO_2_ reagent.

### Trypan-Blue Staining

The dead cells were visually detected using a trypan-blue staining method as described by Liang et al. (2015) with some modifications. Leaves were detached and stained with lactophenol-trypan blue solution (10 mL of lactic acid, 10 mL of glycerol, 10 g of phenol, 10 mg of trypan blue, dissolved in 10 mL of distilled water) at 70°C for 1 h and then boiled for approximately 5 min and left staining overnight. After destaining in chloral hydrate solution (2.5 g of chloral hydrate dissolved in 1 mL of distilled water) for 3 days to reduce background staining, samples were equilibrated with 70% glycerol for scanning.

### Enzyme Extraction and Assay

Fresh leaves (0.2 g) were homogenized with 2 mL of ice-cold 50 mM phosphate buffer (pH 7.8) containing 0.2 mM Na_2_EDTA, 2 mM ascorbate and 2% (m/v) polyvinylpyrrolidone using a chilled mortar and pestle. The homogenate was centrifuged at 12000 *g* for 15 min at 4°C. The supernatant was used for assays of specific enzymatic activities. The activity of SOD was assessed according to Giannopolitis and Ries (1977) by assay its ability to inhibit the photochemical reduction of NBT. One unit of SOD activity was defined as the amount of enzyme that caused 50% inhibition of NBT reduction. The analysis of POD activity was based on the oxidation of guaiacol using H_2_O_2_ according to the method as described by Zhang and Kirham (1994). The CAT activity was measured as decline in absorbance at 240 nm due to the consumption of H_2_O_2_ according to the method of Hamurcu et al. (2013).

### Determination of Proline, Reduced and Oxidized Glutathione (GSH and GSSG) and Soluble Sugars Contents

Proline was extracted and determined by the method described by Mahdavian et al. (2016) with a minor modification. 0.5 g of fresh leaves were homogenized 5 mL 3% (m/v) sulphosalicylic acid and the homogenate was centrifuged at 5000 *g* for 10 min. 2 mL supernatant was added to 2 mL glacial acetic acid and 2 mL acid ninhydrin. The mixture was incubated in a boiling water bath for 1 h and terminated in an ice bath. The reaction mixture was then extracted with 4 mL of toluene with vigorous stirring. The upper toluene phase was separated and an absorbance was recorded at 520 nm. The proline content was calculated using a standard curve and expressed as μmol g^-1^ FW.

Reduced glutathione (GSH) content was determined using the method of Kraj (2016) with some modifications. 0.5 g samples were extracted in an ice bath with 5 mL 100 mM potassium phosphate buffer (pH 7.5) containing 5 mM EDTA, and then centrifuged at 12000 *g* for 20 min. Two milliliter supernatant was mixed with 1 mL 100 mM phosphate buffer (pH 7.5) and 0.5 mL 4 mM DTNB (5,5′-dithio-bisnitrobenzoic acid). The reaction mixture was incubated at 25°C for 10 min, and the absorbance was measured at 412 nm. For GSSG assay, the GSH in supernatant was cleared first, and GSSG content was quantified as described by Knörzer et al. (1996). The GSH and GSSG content was calculated according to their standard curves and expressed as μmol g^-1^ (FW).

Soluble sugar content was measured based on the anthrone method (Azarmi et al., 2016). Fresh leaf samples (0.5 g) were homogenized with 5 mL of 80% ethanol, and the homogenate was centrifuged at 5000 *g* for 15 min. Then, 0.1 mL of the supermatant was mixed with 3 mL of anthrone. The reaction mixture was placed in a boiling water bath for 30 min and then cooled on ice. Absorbance was recorded at 625 nm. The concentration of soluble sugars was calculated using a glucose standard curve and exhibited as mg g^-1^ FW.

### Measurement of Photosynthetic Parameters and Chlorophyll Fluorescence

The photosynthetic rate (*P_n_*), stomatal conductance (*G_s_*), intercellular CO_2_ (*C_i_*) and water use efficiency (WUE) of leaves were measured using a potable photosynthesis system (GSF-3000, Heinz-Walz Instruments, Effeltrich, Germany). Intact leaves were measured at a temperature of 25°C, a photosynthetically active radiation (PAR) of 750 μmol m^-2^ s^-1^, a CO_2_ concentration of 400 μmol mol^-1^ and relative humidity between 40 and 60%.

A modulated imaging chlorophyll fluorometer (IMAGING-PAM M-Series, Heinz-Walz Instruments, Effeltrich, Germany) was used to measure chlorophyll fluorescence parameters according to the instructions provided by the manufacturer. After dark adaptation for 30 min, the fluorescence (F0), maximum fluorescence (Fm) and the non-photochemical quenching (NPQ) were determined (Zhou et al., 2010). The maximal quantum yield of PSII photochemistry in the dark-adapted state was calculated as Fv/Fm.

### Protein Analyses

Thylakoid membrane proteins were isolated as described by Fristedt et al. (2011). Denaturing 12% (w/v) polyacrylamide containing SDS was prepared by the method of Aro et al. (1993). Western blotting analysis was performed according to Chen et al. (2009). Antibodies used (all raised in rabbits) were anti-Arabidopsis D1, D2, CP43, LHCb1, LHCb2, LHCb3 and ACTIN (against 100 amino acids of recombinant actin conserved peptide). These antibodies were purchased from Agrisera Comp. (Umea, Sweden).

### Statistical Analysis

All experiments were repeated at least three times, and mean values were presented with standard deviations (SDs; *n* = 3). Duncan’s multiplication range test was used for comparison among different treatments. The difference was considered to be statistically significant when *P* < 0.05.

## Results

**FIGURE 1:**
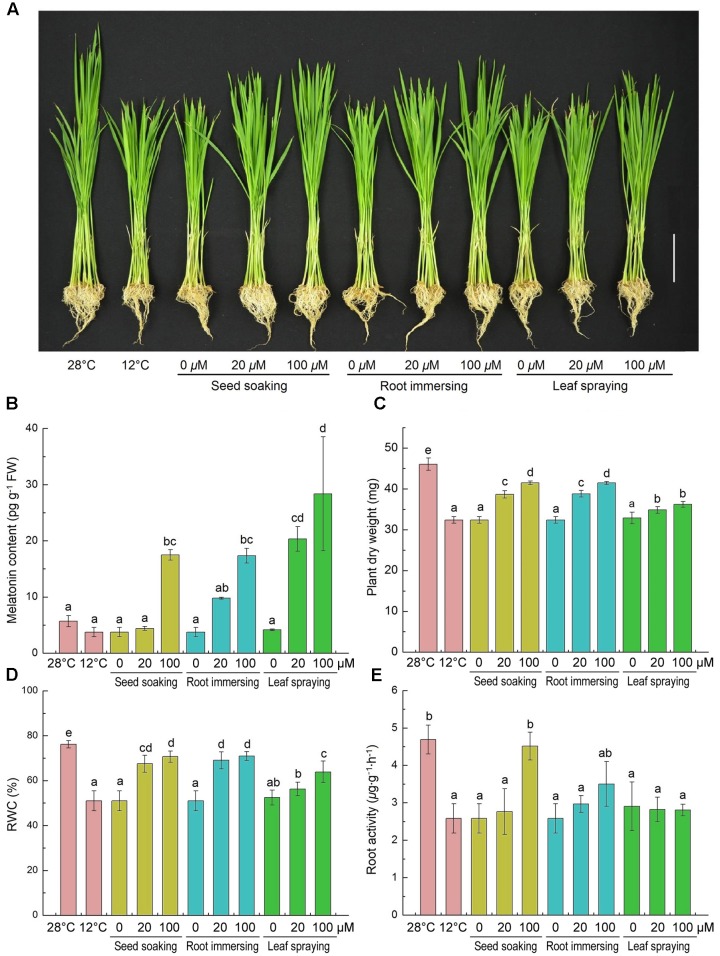
**Effects of exogenous melatonin on growth** **(A)**, melatonin content **(B)**, plant dry weight **(C)**, leaf relative water content **(D)** and root activity **(E)** after 6-day cold stress (12°C). **(A)** Bar = 5 cm. **(B–E)** Data represent means ± SDs of three replicate samples. Bars with different letters indicate significant differences according to Duncan’s multiple range test at *P* < 0.05. I – seed soaking, II – root immersion, III – leaf spraying.

Effects of cold stress and exogenous melatonin on growth of rice seedlings are shown in **Figure 1**. The low temperature stress inhibited the growth of rice seedlings, while melatonin pretreatment alleviated this inhibition to a certain extent (**Figure 1A**). And 100 μM melatonin pretreated plants with higher endogenous melatonin levels grew better than that of 20 μM melatonin pretreated plants (**Figures 1A,B**). Plant DM, RWC and root activity were significantly decreased by cold treatment compared with the control (**Figures 1C–E**). Consistently, melatonin pretreated rice seedlings exhibited significantly greater dry weight and higher RWC than control (**Figures 1C,D**). Seed soaking and root immersing methods showed better protection from cold stress than leaf spraying methods (**Figures 1C,D**). However, only Seed soaking methods in the 100 μM melatonin showed a significant increase in root activity (**Figure 1E**). These results indicate that exogenous melatonin application improved cold stress resistance in rice seedlings.

**FIGURE 2:**
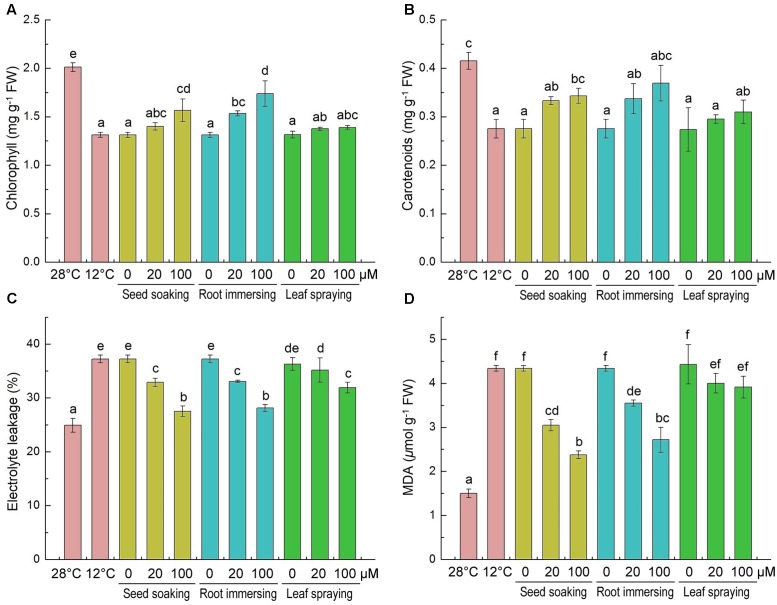
**Effects of exogenous melatonin on chlorophyll content** **(A)**, carotenoids content **(B)**, electrolyte leakage **(C)** and malondialdehyde **(D)** after 6-day cold stress (12°C). Others are the same as in **Figure 1**.

Chlorophyll and carotenoid contents significantly decreased after 6-day cold stress (**Figures 2A,B**), and the decline of pigments caused by the cold stress was mitigated by melatonin application. The chlorophyll and carotenoid contents in melatonin-pretreated seedlings were higher than plants without melatonin pretreatment. The chlorophyll content of 100 μM melatonin pretreated plants was higher than the plants that treated with 20 μM melatonin with root immersing methods (**Figure 2A**). For carotenoid contents, there was no significant difference among the three methods.

The accumulations of MDA and the EL of leaf tissues are markers of lipid peroxidation and damage of cellular membranes. The content of MDA and EL increased significantly in response to cold treatment (**Figures 2C,D**). While the melatonin pretreatment significantly lowered MDA concentration and EL. And the 100 μM melatonin pretreatment resulted in lower MDA content and EL than the 20 μM melatonin pretreatment. These results indicate that melatonin effectively protected the membrane system, and seed soaking and root immersing methods provided better protective effect on membrane system against cold stress (**Figures 2C,D**).

**FIGURE 3:**
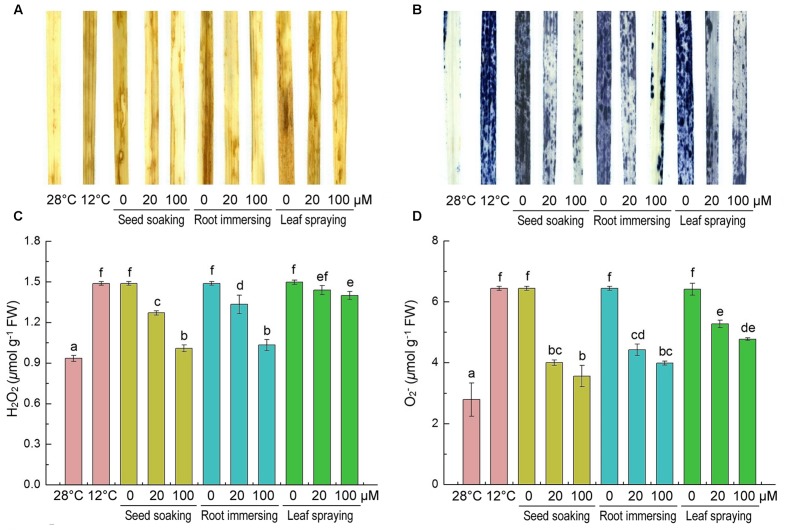
**Effects of exogenous melatonin on H_2_O_2_** **(A,C)**, and $O_{2}^{-}$ **(B,D)** after 6-day cold stress (12°C). Histochemical detection **(A,B)** and content **(C,D)**. Others are the same as in **Figure 1**.

H_2_O_2_ and $O_{2}^{-}$ are two major ROS induced by environmental stresses (Chen et al., 2016). Slight H_2_O_2_ and $O_{2}^{-}$ staining was observed in the control leaves and intensive staining in the cold-treated plants was observed (**Figures 3A,B**). Histochemical staining showed that the H_2_O_2_ and $O_{2}^{-}$ accumulations were alleviated by melatonin application during cold stress. To validate the results of histochemical staining, we measured H_2_O_2_ and $O_{2}^{-}$ contents at the same time. H_2_O_2_ and $O_{2}^{-}$ were extensively accumulated under cold treatment, but they were lower in melatonin-pretreated plants than the control seedlings (**Figures 3C,D**). By seed soaking and root immersing methods of melatonin application, the 100 μM melatonin pretreatment resulted in lower H_2_O_2_ content than 20 μM melatonin pretreatment. These results also suggested that the methods of seed soaking and root immersion could prevent the accumulation of ROS in leaf tissue more effectively than the method of leaf spraying (**Figure 3**).

**FIGURE 4:**
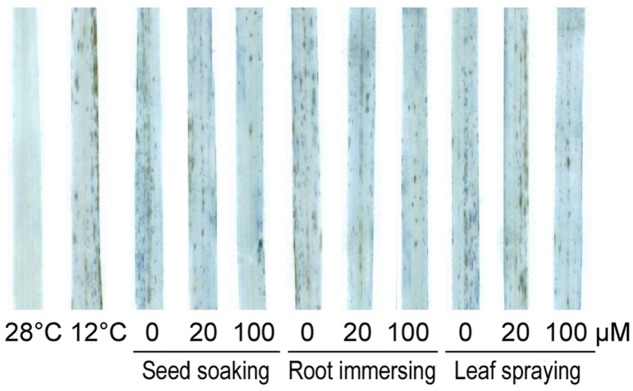
**Trypan-blue staining of control and 6-day cold treatment seedlings.** Others are the same as in **Figure 1**.

We also detected cell death under cold stress with or without melatonin application by trypan-blue staining (**Figure 4**). After cold stress for 6 days, obvious cell death occurred in rice seedlings in comparison with non-stressed plants. While, the cell dead was significantly alleviated in melatonin pretreated seedlings. The protective effect of 100 μM melatonin pretreatment was more significant than 20 μM melatonin pretreatment.

**FIGURE 5:**
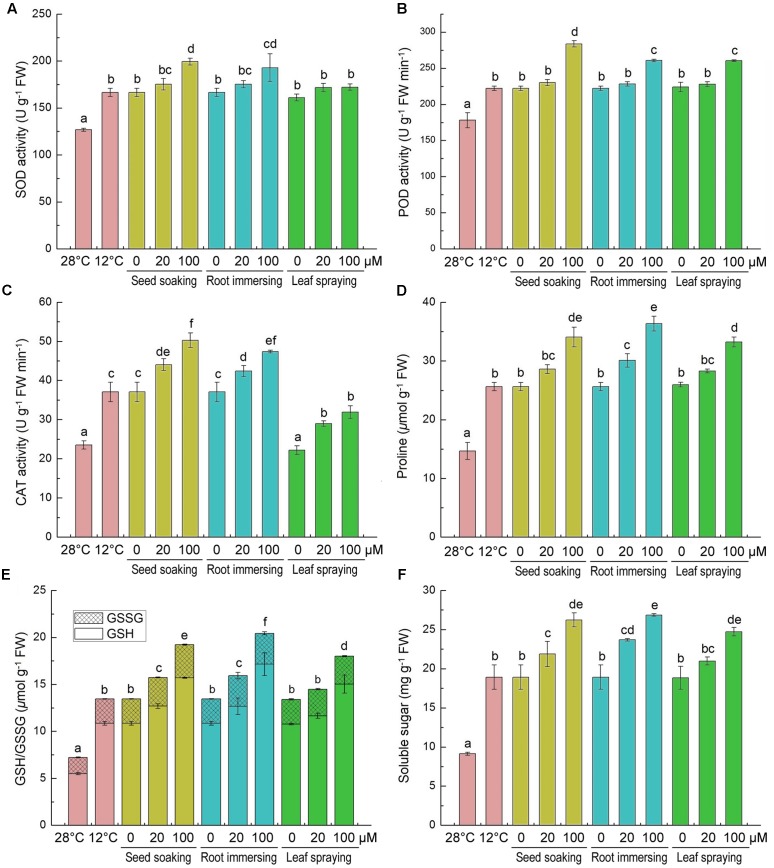
**Effects of exogenous melatonin on SOD activity** **(A)**, POD activity **(B)**, CAT activity **(C)**, proline content **(D)**, GSH content **(E)**, and soluble sugar content **(F)** after 6-day cold stress (12°C). Others are the same as in **Figure 1**.

The effects of cold stress and melatonin applications on specific activities of antioxidant enzymes and on non-enzymatic antioxidants in rice seedlings are showed in **Figure 5**. The activities of antioxidant enzyme (SOD, CAT, and POD) were greatly induced by the cold stress (**Figures 5A–C**). Additionally, melatonin pretreated plants showed more higher activities of antioxidant enzymes (SOD, CAT, and POD), especially when the concentrations of melatonin were 100 μM (**Figures 5A–C**). The three different methods of melatonin application did not show significant differences in SOD and POD activities (**Figures 5A,B**), while the seed soaking and root immersion caused more increasement of CAT activity (**Figures 5C**).

We also measured the contents of proline, GSH/GSSG and soluble sugar, because they are important non-enzymatic antioxidants. After cold stress for 6 days, compared with the control, the contents of proline, GSH, GSSG and soluble sugar were significantly increased (**Figures 5D–F**). Moreover, the melatonin application further increased their accumulation under cold stress conditions. Meanwhile, compared to the cold treated seedlings alone, the 100 μM melatonin pretreatment increased the proline, GSH and soluble sugar more than the 20 μM melatonin pretreatment. Besides, the effects of three melatonin-application methods showed no significant difference (**Figures 5D–F**).

**FIGURE 6:**
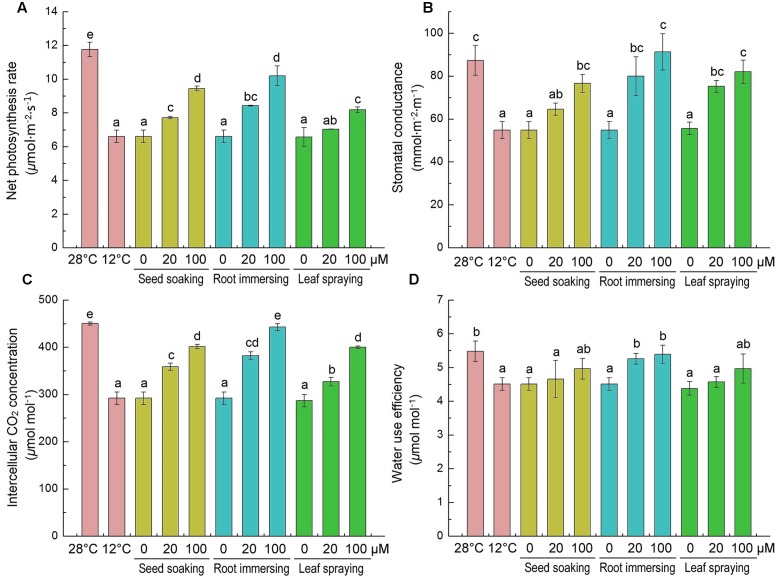
**Effects of exogenous melatonin on net photosynthetic rate (*P_n_*)** **(A)**, stomatal conductance (*G_s_*) **(B)**, intercellular CO_2_ (*C_i_*) **(C)** and water use efficiency (WUE) **(D)** after 6-day cold stress (12°C). Others are the same as in **Figure 1**.

The effects of cold stress and melatonin application on photosynthesis were evaluated by monitoring gas exchange parameters [net photosynthetic rate (*P_n_*), stomatal conductance (*G_s_*), intercellular CO_2_ (*C_i_*) and WUE]. Cold stress significantly decreased all of these parameters, as shown in **Figures 6**. These parameters were higher in melatonin pretreated rice seedlings than the control plants. Melatonin caused significant increases in net photosynthetic rate, stomatal conductance and intercellular CO_2_ (**Figures 6A–C**), especially in the group of 100 μM melatonin pretreatment by root immersing method. While, only the root immersing method caused a significant increase in water use efficiency (**Figure 6D**).

**FIGURE 7:**
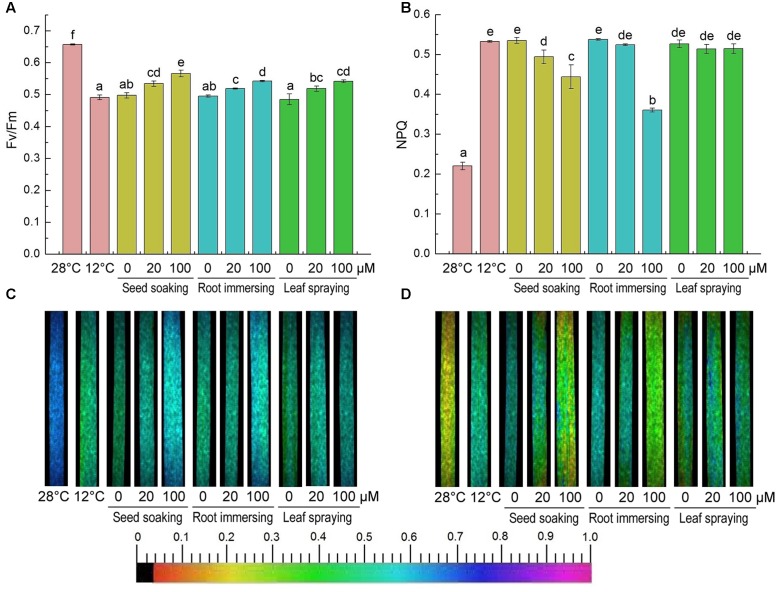
**Effects of exogenous melatonin on maximum PSII quantum yield (Fv/Fm)** **(A,C)**, and non-photochemical quenching (NPQ) **(B,D)** after 6-day cold stress (12°C). Quantitative values **(A,B)** and fluorescence image **(C,D)**. Others are the same as in **Figure 1**.

After cold treatment, the maximum photochemical efficiency of PSII (Fv/Fm) was significantly decreased, but it was higher in melatonin pretreated plants (**Figures 7A,C**). And there were no significant differences among the three melatonin-application methods. In contrast to the change of Fv/Fm, the non-photochemical quenching (NPQ) was significantly increased after 6-day cold stress (**Figures 7B,D**). This increase was partly reversed by melatonin application (**Figures 7B,D**). The chlorophyll fluorescence data suggested that the seed soaking and root immersing applications were more effective than the leaf spraying (**Figure 7**).

**FIGURE 8:**
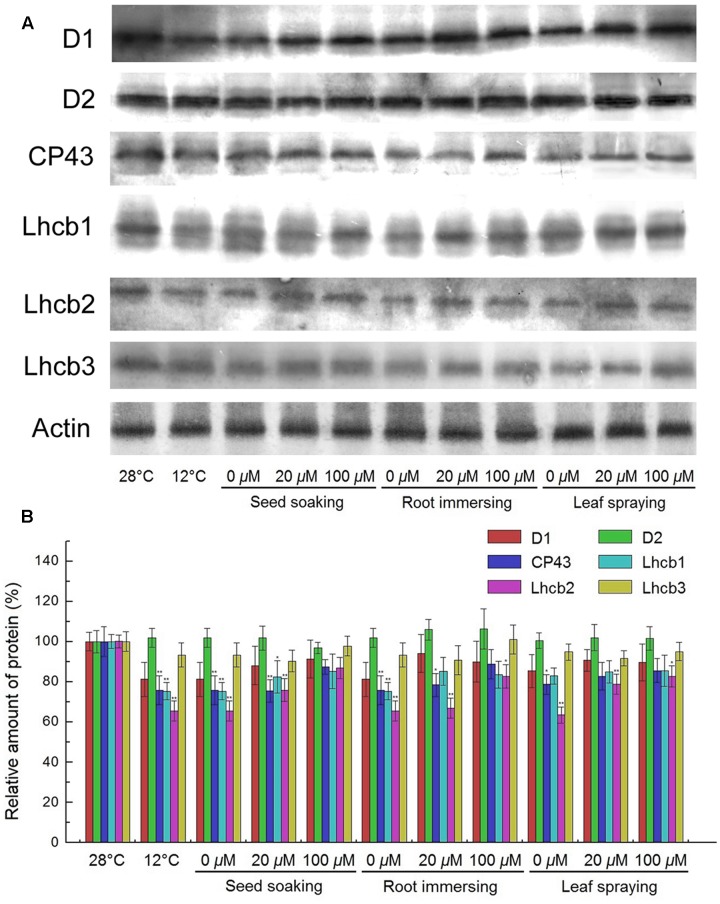
**Effects of exogenous melatonin on thylakoid membrane proteins.** **(A)** Immunoblotting was performed with antibodies against D1, D2, CP43, Lhcb1, Lhcb2, Lhcb3 and Actin conserved peptide. Loading was according to an equal amount of chlorophyll. Results by SDS-PAGE after blue staining (CBS) are shown in the bottom panel. **(B)** The quantification of immunoblot data. Results are relative to the amount in the 28°C (100%). ^∗,^^∗∗^ indicate statistically significant differences at *P* < 0.05 and *P* < 0.01, respectively, determined using Duncan’s multiple range test. Others are the same as in **Figure 1**.

PSII proteins were further detected by Western blotting. The results showed that the contents of D1, CP43, Lhcb1, and Lhcb2 were significantly reduced after exposure to cold conditions, while the contents of D2 and Lhcb3 proteins showed no significant change (**Figure 8**). However, higher D1, CP43, Lhcb1, and Lhcb2 contents were found for the melatonin pretreated plants, compared with the seedlings without melatonin treatments. For these PSII proteins, seedlings of 100 μM melatonin pretreatment showed better protective effects than those of the 20 μM melatonin pretreatment. While the three methods did not show any significant difference.

## Discussion

Low temperature stress critically inhibits plant growth. In the present study, we found that the growth of rice seedlings was significantly inhibited under low temperature conditions. However, the application of exogenous melatonin significantly alleviated the cold-stress-induced growth inhibition. These results are in agreement with the previous reports that melatonin application can enhance cold tolerance of *Arabidopsis* (Bajwa et al., 2014), wheat (Hulya et al., 2014), and bermudagrass (Hu et al., 2016). In the present study, we also investigated the effects of three different methods of melatonin application and our results showed the seed soaking and root immersion was more effective than leaf spraying. However, quantitative analysis of melatonin showed that the melatonin content of the leaf-spraying method was higher than the other two methods, especially in the plants treated with100 μM melatonin. We presume that large amounts of melatonin may stay on the leaf surface and only a few was absorbed into the leaf tissue when melatonin was sprayed to the leaves.

Photosynthetic pigments are susceptible to environmental stresses (Ashraf and Harris, 2013). Chlorophylls, the most abundant and essential pigments in higher plants, responsible for capturing light energy and driving electrons to the reaction center during photosynthesis. Under biotic or abiotic stresses, seedlings have decreased chlorophyll contents due to impaired chlorophyll biosynthesis or accelerated chlorophyll degradation (Perveen et al., 2010). In the present study, we showed that chlorophylls and carotenoids were significantly decreased when the seedlings were exposed to cold stresses. However, this inhibitory effect of cold stress on pigments was alleviated by melatonin pretreatments, and they showed some dose-dependent response. A high concentration of 100 μM melatonin provided better protective effects than the 20 μM treatment. These results indicate that exogenous melatonin pre-treatment improved cold stress tolerance in rice seedlings.

Lipid peroxidation and the subsequent increase in MDA content are reported in previous studies during cold stress (Hulya et al., 2014; Hu et al., 2016). EL is a good indicator of the permeability of the plasma membrane and increased under stress conditions (Liu et al., 2015; Zeng et al., 2015). Compare with the control seedlings, the cold-stressed seedlings showed a 180% increase in MDA content, and a 49% increase in EL (**Figures 2C,D**). These findings are consistent with prior researchers that abiotic stress markedly enhanced MDA content and EL in plants (Hulya et al., 2014; Liu et al., 2015).

Reactive oxygen species are the byproducts of photosynthesis, respiration and other normal metabolic processes, and they play an important role in stress tolerance (Chen et al., 2016). ROS are also involved in programmed cell death, abiotic stress response and virus defense (Shi et al., 2015a,b,c). However, excessive accumulation of ROS can stimulate membrane lipid peroxidation, and then lead to the damage of cell membrane, loss of cellular integrity, and cell death (Chen, 2003; Zeng et al., 2015). In the present study, $O_{2}^{-}$ and H_2_O_2_ increased significantly under cold stress conditions. However, pretreatments with melatonin significantly decreased ROS levels in leaf tissues, and the alleviatory effects were more obvious for the seed soaking and root immersion methods than the leaf spraying method.

Plants have evolved enzymatic antioxidant system or the non-enzymatic antioxidant system to maintain the redox balance. Among enzymatic antioxidant defense systems, SOD is a key enzyme that dismutates $O_{2}^{-}$ to O_2_ and H_2_O_2_, while CAT and POD can break down H_2_O_2_ to H_2_O through different pathway in plant cells (Mittler, 2002; Hu et al., 2016). While the non-enzymatic antioxidant system plays an important role in maintaining normal cell metabolism and increasing plant tolerance to stresses (Liu et al., 2015). Melatonin is a broad spectrum antioxidant and a crucial radical scavenger and could stimulate the activities of antioxidant enzymes (Hulya et al., 2014; Hu et al., 2016), and exogenous melatonin application might activate enzymatic antioxidant system or the non-enzymatic antioxidant system to maintain the redox balance. Shi et al. (2015a) found that exogenous melatonin alleviated ROS accumulation and cold-induced oxidative damages by directly scavenging ROS and enhancing antioxidative enzymes in bermudagrass. In present study, melatonin pretreatments led to increasing in SOD, POD and CAT activities of rice seedlings. GSH is the main component of non-enzymatic antioxidant system in plants which is involved in direct and indirect regulations to ROS levels (Ning et al., 2015). GSH regulates the redox homeostasis of cell together with its oxidized form (GSSG) and based on the reduction potential of the GSH/GSSG couple (Szalai et al., 2009). Both of GSH and GSSG contents increased under the cold stress in this study, which could be further promoted by melatonin applications (**Figures 5E**). These results indicated that melatonin applications might promote GSH accumulation in plants suffered from cold stress.

Photosynthesis is an important physiological process occurring in green plants, which is the basis of the biological world, and is also an important medium for the earth’s carbon and oxygen cycle. It is well known that photosynthesis is very sensitive to low temperatures (Dalal and Tripathy, 2012), which could induced the decrease of photosynthetic pigments, destruction of chloroplasts structure, and stomatal closure, and so forth (Cai et al., 2016). In the present study, rice seedlings experienced a pronounced depression in *P_n_* under low temperature (**Figure 6A**), and this phenomenon has been also reported in other stress conditions (Wang et al., 2013a; Cai et al., 2016; Li et al., 2017). We observed that the decreased *P_n_* was accompanied by significant declines in *Gs* and *C_i_* (**Figures 6B,C**). Therefore, we presume that decrease of *P_n_* under low temperature conditions may be attributed to stomatal limitation. In addition, both stomatal and non-stomatal inhibitions could reduce *P_n_*. The decrease in stomatal conductance could result in a declined *P_n_* and reduced assimilation products, thus causing an inhibited growth and a lower yield (Easwar Rao and Chaitanya, 2016). Melatonin applications can alleviate cold-stress-induced inhibitions to photosynthetic activities, and the seed soaking and root immersion applications were more effective than leaf spraying.

Chlorophyll fluorescence analysis was one of the most powerful and widely used means for obtaining the functional status of PSII (Wang et al., 2013b), and the Fv/Fm ratio represents the maximal photochemical efficiency of PSII (Zhou et al., 2010). Fv/Fm decreased because of photoinhibition or PSII damages. While the thermal dissipation, represented by NPQ, increased (Wang et al., 2013a; Zhang et al., 2013). Under the cold stress, the value of Fv/Fm decreased significantly, but it was still higher in melatonin pretreated plants, and NPQ was also kept at a high value. Melatonin treated rice seedlings maintained lower NPQ, also suggesting that melatonin could protect photosynthetic system from cold-stress damages. Similar changes were also observed in other abiotic stresses (Wang et al., 2013a; Zhang et al., 2013). For chlorophyll fluorescence, we did not find any significant difference among the three methods of melatonin application, while the decrease of NPQ was more obvious in the seed soaking method than the other two methods.

PSII is a multi-subunit pigment-protein complex that catalyzes the light-driven electron transfer from water to plastoquinone (PQ) in the thylakoid membrane of high plants, algae, and cyanobacteria (Baena-González et al., 2013). PSII plays an especially important role in the responses of photosynthesis in the higher plants to environment stresses (Yuan et al., 2005). Previous studies have shown that D1 protein, one of the core proteins of PSII, is the most vulnerable component in PSII reaction center under stress conditions (Luo et al., 2009; Li et al., 2017). It has been shown that the D1 protein can be significantly damaged by environmental stresses (Luo et al., 2009). In the present study, the lighter decreases in PSII proteins indicated the protective role of melatonin to PSII under cold stress.

In summary, the applications of exogenous melatonin alleviated the cold-stress-induced inhibition to plant growth by enhancing the activities of antioxidative enzymes and the levels of non-enzyme antioxidants. Melatonin could protect photosynthetic system from oxidative damages. The melatonin application by the seed soaking and root immersion was more effective than the leaf spraying method. All the effects of melatonin depended on its concentrations and the application methods. Our results imply that melatonin could be considered as an utilizable substance to improve plant cold tolerance.

## Author Contributions

SY and MY designed the experiments; Q-HH, BH, C-BD, Z-WZ, Y-EC, CH, L-JZ, YH, and J-QL performed the experiments and data analysis; and Q-HH, SY, and MY wrote the manuscript.

## Conflict of Interest Statement

The authors declare that the research was conducted in the absence of any commercial or financial relationships that could be construed as a potential conflict of interest.
